# Supplementary material for: Medical students’ perceptions and motivations during the COVID-19 pandemic
Source: PLoS One. 2021 Mar 17;16(3):e0248627. doi: 10.1371/journal.pone.0248627 (PMC7968644; doi:10.1371/journal.pone.0248627)
Supplement: S1 Table — (DOCX) [file pone.0248627.s001.docx]

**S1 Table. Distribution of answers by each statement for all participants (n = 10,433).**

|  | Agree | Neither agree nor disagree | Disagree |
| --- | --- | --- | --- |
| S1 I feel prepared to identify a patient with suspected infection | 6393 (61.3) | 2117 (20.3) | 1923 (18.4) |
| S2 I can identify signs of severity in a patient | 7650 (73.3) | 1504 (14.4) | 1279 (12.3) |
| S3 I know how to guide patients in preventive measures | 9967 (95.5) | 317 (3.0) | 149 (1.4) |
| S4 I know how to guide patients in therapeutic measures | 3260 (31.2) | 2862 (27.2) | 4311 (41.3) |
| S5 I know how to use personal protection equipment (PFE) | 8255 (79.1) | 1366 (13.1) | 812 (7.8) |
| S6 I am able to participate in the care of patients who seek health care | 3449 (33.1) | 2757 (26.4) | 4227 (40.5) |
| S7 I feel able to communicate a diagnosis of COVID-19 infection | 4001 (38.4) | 2373 (22.7) | 4049 (38.8) |
| S8 Medical internship students must participate in health care assistance during pandemic | 6290 (60.3) | 2619 (25.1) | 1524 (14.6) |
| S9 All students, regardless of their year in medical school, must participate in health care assistance during pandemic | 1398 (13.4) | 2132 (20.4) | 6903 (66.2) |
| S10 It is the duty of the medical student to put himself or herself at the service of the population in the pandemic | 4460 (42.9) | 3073 (29.5) | 2884 (27.6) |
| S11 I feel insecure regarding the future | 6769 (64.9) | 1870 (17.9) | 1794 (17.2) |
| S12 I am afraid of contaminating myself | 6433 (61.7) | 2020 (19.4) | 1980 (19.0) |
| S13 Medical schools must suspend their academic activities during the first to fourth years. | 8125 (77.9) | 1393 (13.4) | 915 (8.8) |
| S14 Medical schools must suspend their academic activities during internships | 3366 (32.3) | 3243 (31.1) | 3824 (36.7) |
| S15 Distance learning must be implemented during the suspension of academic activities | 6142 (58.9) | 1982 (19.0) | 2309 (22.1) |
| S16 I would prefer to delay my training to fully replace academic activities than to participate in distance learning activities | 3519 (33.7) | 2008 (19.2) | 4906 (47.0) |
| S17 After the pandemic, academic activities must be fully resumed | 6112 (58.6) | 2280 (21.9) | 2041 (19.6) |
| S18 After the pandemic, only practical academic activities must be resumed | 4349 (41.7) | 2052 (19.7) | 4032 (38.6) |
| S19 I feel able to study my medical course content through distance learning | 5278 (50.6) | 2292 (22.0) | 2863 (27.4) |
| S20 I prefer to study theoretical content using distance learning methods | 4557 (43.7) | 2304 (22.1) | 3572 (34.2) |
| S21 My emotional state during the pandemic affects my learning | 4264 (40.9) | 2269 (21.7) | 3900 (37.4) |
| S22 I will be a better health professional for having experienced the pandemic | 5934 (56.9) | 3124 (29.9) | 1375 (13.2) |
| S23 I feel stressed in the hospital at the moment | 3609 (34.6) | 3717 (35.6) | 3107 (29.8) |
| S24 The supervision I receive in my practice fields is good | 4811 (46.8) | 3956 (37.9) | 1596 (15.3) |
| S25 I have access to psychological support | 4379 (42.0) | 2192 (21.0) | 3862 (37.0) |
| S26 I am proud of the way my institution responded to social and health demands in the face of the pandemic | 5549 (53.2) | 2781 (26.7) | 2103 (20.2) |
| S27 The role of medical students during the pandemic is irrelevant | 664 (6.4) | 1951 (18.7) | 7818 (74.9) |
| S28 I am willing to take risks by participating in practice in the context of the pandemic | 4830 (46.3) | 2352 (22.5) | 3251 (31.2) |
