# Supplementary material for: Medical students’ perceptions and motivations during the COVID-19 pandemic
Source: PLoS One. 2021 Mar 17;16(3):e0248627. doi: 10.1371/journal.pone.0248627 (PMC7968644; doi:10.1371/journal.pone.0248627)
Supplement: S2 Table — Students were divided into three groups according to their opinions about the participation of medical students in the care of patients with COVID-19. The number and percentage of medical students who answered “completely agree” or “agree” to each of the statements is presented. (DOCX) [file pone.0248627.s002.docx]

**S2 Table. Student characteristics and perceptions according to their views about the role of medical students during the COVID-19 pandemic. Students were divided into three groups according to their opinions about the participation of medical students in the care of patients with COVID-19. The number and percentage of medical students who answered “completely agree” or “agree” to each of the statements is presented.**

|  | No students should participate  (N=4072) | Only students in internships should participate  (N=4963) | All students should participate  (N=1398) | Total  (N=10433) | |
| --- | --- | --- | --- | --- | --- |
| Students’ characteristics | | | | |  |
| Age (mean ± SD) | 22.7 ± 3.7 | 22.4 ± 3.9 | 22.4 ± 4.2 | 22.5 ± 3.9 | |
| Female sex (N (%)) | 3008 (73.9%) | 3371 (67.9%) | 888 (63.5%) | 7267 (69.7%) | |
| Personal/family/friend diagnosed with COVID-19 (N (%)) | 308 (7.6%) | 334 (6.7%) | 85 (6.1%) | 727 (7.0%) | |
| Year in medical school (N (%))  First/second year (Basic sciences)  Third/fourth (Clinical sciences)  Fifth/sixth (Internship) | 1472 (36.1%)  1391 (34.2%)  1209 (29.7%) | 2265 (45.6%)  1552 (31.3%)  1146 (23.1%) | 830 (59.4%)  478 (34.2%)  90 (6.4%) | 4567 (43.8%)  3421 (32.8%)  2445 (23.4%) | |
| Students’ perceptions | | | | | |
| S3. I know how to guide patients in preventive measures | 3805 (93.4%) | 4811 (96.9%) | 1351 (96.6%) | 9967 (95.5%) | |
| S5. I know how to use personal protection equipment (PFE) | 2988 (73.4%) | 4068 (82.0%) | 1199 (85.8%) | 8255 (79.1%) | |
| S13. Medical schools must suspend their academic activities during the first to fourth years. | 3436 (84.4%) | 3925 (79.1%) | 764 (54.6%) | 8125 (77.9%) | |
| S2. I can identify signs of severity in a patient | 2853 (70.1%) | 3701 (74.6%) | 1096 (78.4%) | 7650 (73.3%) | |
| S11. I feel insecure regarding the future | 2835 (69.6%) | 3117 (62.8%) | 817 (58.4%) | 6769 (64.9%) | |
| S12. I am afraid of contaminating myself | 2937 (72.1%) | 2829 (57.0%) | 667 (47.7%) | 6433 (61.7%) | |
| S1. I feel prepared to identify a patient with suspected infection | 2270 (55.7%) | 3173 (63.9%) | 950 (68.0%) | 6393 (61.3%) | |
| S15. Distance learning must be implemented during the suspension of academic activities | 2446 (60.1%) | 2899 (58.4%) | 797 (57.0%) | 6142 (58.9%) | |
| S17. After the pandemic, academic activities must be fully resumed | 2262 (55.6%) | 2913 (58.7%) | 937 (67.0%) | 6112 (58.6%) | |
| S22. I will be a better health professional for having experienced the pandemic | 1807 (44.4%) | 3105 (62.6%) | 1022 (73.1%) | 5934 (56.9%) | |
| S26. I am proud of the way my institution responded to social and health demands in the face of the pandemic | 1958 (48.1%) | 2758 (55.6%) | 833 (59.6%) | 5549 (53.2%) | |
| S19. I feel able to study my medical course content through distance learning | 2040 (50.1%) | 2533 (51.0%) | 705 (50.4%) | 5278 (50.6%) | |
| S24. The supervision I receive in my practice fields is good | 1540 (37.8%) | 2495 (50.3%) | 846 (60.5%) | 4881 (46.8%) | |
| S28. I am willing to take risks by participating in practice in the context of the pandemic | 904 (22.2%) | 2785 (56.1%) | 1141 (81.6%) | 4830 (46.3%) | |
| S20. I prefer to study theoretical content using distance learning methods | 1810 (44.4%) | 2181 (43.9%) | 566 (40.5%) | 4557 (43.7%) | |
| S10. It is the duty of the medical student to put himself or herself at the service of the population in the pandemic | 691 (17.0%) | 2525 (50.9%) | 1260 (90.1%) | 4476 (42.9%) | |
| S25. I have access to psychological support | 1384 (34.0%) | 2212 (44.6%) | 783 (56.0%) | 4379 (42.0%) | |
| S18. After the pandemic, only practical academic activities must be resumed | 1658 (40.7%) | 2075 (41.8%) | 616 (44.1%) | 4349 (41.7%) | |
| S21. My emotional state during the pandemic affects my learning | 1939 (47.6%) | 1828 (36.8%) | 497 (35.6%) | 4264 (40.9%) | |
| S7. I feel able to communicate a diagnosis of COVID-19 infection | 1167 (28.7%) | 2062 (41.5%) | 782 (55.9%) | 4011 (38.4%) | |
| S23. I feel stressed in the hospital at the moment | 1974 (48.5%) | 1371 (27.6%) | 264 (18.9%) | 3609 (34.6%) | |
| S16. I would prefer to delay my training to fully replace academic activities than to participate in distance learning activities | 1373 (33.7%) | 1602 (32.3%) | 544 (38.9%) | 3519 (33.7%) | |
| S6. I am able to participate in the care of patients who seek health care | 807 (19.8%) | 1891 (38.1%) | 751 (53.7%) | 3449 (33.1%) | |
| S14. Medical schools must suspend their academic activities during internships | 2236 (54.9%) | 832 (16.8%) | 298 (21.3%) | 3366 (32.3%) | |
| S4. I know how to guide patients in therapeutic measures | 1007 (24.7%) | 1689 (34.0%) | 564 (40.3%) | 3260 (31.2%) | |
| S27. The role of medical students during the pandemic is irrelevant | 444 (10.9%) | 144 (2.9%) | 76 (5.4%) | 664 (6.4%) | |
