# Supplementary material for: Medical students’ perceptions and motivations during the COVID-19 pandemic
Source: PLoS One. 2021 Mar 17;16(3):e0248627. doi: 10.1371/journal.pone.0248627 (PMC7968644; doi:10.1371/journal.pone.0248627)
Supplement: S3 Table — (DOCX) [file pone.0248627.s003.docx]

**S3 Table: Relevant items in exploratory factor analysis scores calculation.**

| **Statement** | **Direction** | **Value** | **Factor** |
| --- | --- | --- | --- |
| **S16 I would prefer to delay my training to fully replace academic activities than to participate in distance learning activities** | Disagree | -0.67 | 1 |
| **S17 After the pandemic, academic activities must be fully resumed** | Disagree | -0.55 | 1 |
| **S18 After the pandemic, only practical academic activities must be resumed** | Agree | 0.50 | 1 |
| **S20 I prefer to study theoretical content using distance learning methods** | Agree | 0,59 | 1 |
| **S19 I feel able to study my medical course content through distance learning** | Agree | 0,65 | 1 |
| **S15 Distance learning must be implemented during the suspension of academic activities** | Agree | 0,73 | 1 |
| **S2 I can identify signs of severity in a patient** | Agree | 0.46 | 2 |
| **S1 I feel prepared to identify a patient with suspected infection** | Agree | 0.49 | 2 |
| **S4 I know how to guide patients in therapeutic measures** | Agree | 0.51 | 2 |
| **S7 I feel able to communicate a diagnosis of COVID-19 infection** | Agree | 0.64 | 2 |
| **S6 I am able to participate in the care of patients who seek health care** | Agree | 0.70 | 2 |
| **S14 Medical schools must suspend their academic activities during internships** | Agree | 0.32 | 3 |
| **S11 I feel insecure regarding the future** | Agree | 0.46 | 3 |
| **S21 My emotional state during the pandemic affects my learning** | Agree | 0.51 | 3 |
| **S23 I feel stressed in the hospital at the moment** | Agree | 0.52 | 3 |
| **S12 I am afraid of contaminating myself** | Agree | 0.55 | 3 |
| **S22 I will be a better health professional for having experienced the pandemic** | Agree | 0.32 | 4 |
| **S28 I am willing to take risks by participating in practice in the context of the pandemic** | Agree | 0,50 | 4 |
| **S10 It is the duty of the medical student to put himself or herself at the service of the population in the pandemic** | Agree | 0.60 | 4 |

Factor 1: Remote learning

Factor 2: Medical knowledge self-efficiency

Factor 3: Psychological stress

Factor 4: Professional values/altruism
