# Supplementary material for: Medical students’ perceptions and motivations during the COVID-19 pandemic
Source: PLoS One. 2021 Mar 17;16(3):e0248627. doi: 10.1371/journal.pone.0248627 (PMC7968644; doi:10.1371/journal.pone.0248627)
Supplement: S4 Table — (DOCX) [file pone.0248627.s004.docx]

**S4 Table: Crude odds ratios (95% confidence intervals) for the association between students’ characteristics and perceptions and their views on the role of medical students during the COVID-19 pandemic**

|  | |  | No students should participate | Only students in internships should participate | All students should participate |
| --- | --- | --- | --- | --- | --- |
|  | Year in medical school | | | | |
| First/second (Basic sciences) | |  | 1.0 (Reference) | Reference | Reference |
| Third/fourth (Clinical sciences) | |  | 1.0 (Reference) | **0.73 (0.66 - 0.80)** | **0.61 (0.53 - 0.70** |
| Fifth/sixth (Internship) | |  | 1.0 (Reference) | **0.62 (0.56 - 0.68)** | **0.13 (0.10 - 0.17)** |
|  | Sex | | | | |
| Female | |  | 1.0 (Reference) | 1.0 (Reference) | 1.0 (Reference) |
| Male | |  | 1.0 (Reference) | **1.34 (1.22 - 1.46)** | **1.62 (1.43 - 1.85)** |
|  | Personal/family/friend diagnosis of COVID-19 | | | | |
| No | |  | 1.0 (Reference) | 1.0 (Reference) | 1.0 (Reference) |
| Yes | |  | 1.0 (Reference) | 0.88 (0.75 - 1.04) | 0.79 (0.62 - 1.01) |
|  | Beliefs in support of the participation of medical students in COVID-19 pandemic healthcare | | | | |
| S10. It is the duty of the medical student to put himself or herself at the service of the population in the pandemic | |  | 1.0 (Reference) | **5.07 (4.59 - 5.59)** | **44.66 (36.79 - 54.21)** |
| S28. I am willing to take risks by participating in practice in the context of the pandemic | |  | 1.0 (Reference) | **4.48 (4.08 - 4.92)** | **15.56 (13.34 - 18.16)** |
| S6. I am able to participate in the care of patients who seek health care | |  | 1.0 (Reference) | **2.49 (2.26 - 2.74)** | **4.70 (4.12 - 5.35)** |
| S22. I will be a better health professional for having experienced the pandemic | |  | 1.0 (Reference) | **2.09 (1.93 - 2.28)** | **3.41 (2.98 - 3.89)** |
| S7. I feel able to communicate a diagnosis of COVID-19 infection | |  | 1.0 (Reference) | **1.77 (1.62 - 1.93)** | **3.16 (2.79 - 3.58)** |
| S24. The supervision I receive in my practice fields is good | |  | 1.0 (Reference) | **1.66 (1.53 - 1.81)** | **2.52 (2.22 - 2.85)** |
| S25. I have access to psychological support | |  | 1.0 (Reference) | **1.56 (1.43 - 1.70)** | **2.47 (2.18 - 2.80)** |
| S5. I know how to use personal protection equipment (PFE) | |  | 1.0 (Reference) | **1.65 (1.49 - 1.82)** | **2.19 (1.85 - 2.58)** |
| S4. I know how to guide patients in therapeutic measures | |  | 1.0 (Reference) | **1.57 (1.43 - 1.72)** | **2.06 (1.81 - 2.34)** |
| S3. I know how to guide patients in preventive measures | |  | 1.0 (Reference) | **2.22 (1.81 - 2.72)** | **2.02 (1.47 - 2.77)** |
| S1. I feel prepared to identify a patient with suspected infection | |  | 1.0 (Reference) | **1.41 (1.29 - 1.53)** | **1.68 (1.48 - 1.91)** |
| S17. After the pandemic, academic activities must be fully resumed | |  | 1.0 (Reference) | **1.14 (1.05 - 1.24)** | **1.63 (1.43 - 1.85)** |
| S26. I am proud of the way my institution responded to social and health demands in the face of the pandemic | |  | 1.0 (Reference) | **1.35 (1.24 - 1.47)** | **1.59 (1.41 - 1.80)** |
| S2. I can identify signs of severity in a patient | |  | 1.0 (Reference) | **1.25 (1.14 - 1.37)** | **1.55 (1.34 - 1.79)** |
| S16. I would prefer to delay my training to fully replace academic activities than to participate in distance learning activities | |  | 1.0 (Reference) | 0.94 (0.86 - 1.02) | **1.25 (1.10 - 1.42)** |
| S18. After the pandemic, only practical academic activities must be resumed | |  | 1.0 (Reference) | 1.05 (0.96 - 1.14) | **1.15 (1.01 - 1.30)** |
|  | Beliefs not/poorly related with the participation of medical students in COVID-19 pandemic healthcare | | | | |
| S19. I feel able to study my medical course content through distance learning | |  | 1.0 (Reference) | 1.04 (0.96 - 1.13) | 1.01 (0.90 - 1.14) |
|  | Beliefs against the participation of medical students in COVID-19 pandemic healthcare | | | | |
| S15. Distance learning must be implemented during the suspension of academic activities | |  | 1.0 (Reference) | 0.93 (0.86 - 1.02) | **0.88 (0.78 - 1.00)** |
| S20. I prefer to study theoretical content using distance learning methods | |  | 1.0 (Reference) | 0.98 (0.90 - 1.07) | **0.85 (0.75 - 0.96)** |
| S11. I feel insecure regarding the future | |  | 1.0 (Reference) | **0.74 (0.67 - 0.80)** | **0.61 (0.54 - 0.70)** |
| S21. My emotional state during the pandemic affects my learning | |  | 1.0 (Reference) | **0.64 (0.59 - 0.70)** | **0.61 (0.54 - 0.69)** |
| S27. The role of medical students during the pandemic is irrelevant | |  | 1.0 (Reference) | **0.24 (0.20 - 0.30)** | **0.47 (0.37 - 0.60)** |
| S12. I am afraid of contaminating myself | |  | 1.0 (Reference) | **0.51 (0.47 - 0.56)** | **0.35 (0.31 - 0.40)** |
| S23. I feel stressed in the hospital at the moment | |  | 1.0 (Reference) | **0.41 (0.37 - 0.44)** | **0.25 (0.21 - 0.29)** |
| S13. Medical schools must suspend their academic activities during the first to fourth years | |  | 1.0 (Reference) | **0.70 (0.63 - 0.78)** | **0.22 (0.19 - 0.26)** |
| S14. Medical schools must suspend their academic activities during internships | |  | 1.0 (Reference) | **0.17 (0.15 - 0.18)** | **0.22 (0.19 - 0.26)** |
