# Supplementary material for: Medical students’ perceptions and motivations during the COVID-19 pandemic
Source: PLoS One. 2021 Mar 17;16(3):e0248627. doi: 10.1371/journal.pone.0248627 (PMC7968644; doi:10.1371/journal.pone.0248627)
Supplement: S5 Table — (DOCX) [file pone.0248627.s005.docx]

**S5 Table: Crude odds ratios (95% confidence intervals) for the association between internship students’ characteristics and perceptions and their views on the role of medical students during the COVID-19 pandemic.**

|  | No students should participate | Only students in internships should participate | All students should participate |
| --- | --- | --- | --- |
| Sex | | | |
| Female | 1.0 (Reference) | 1.0 (Reference) | 1.0 (Reference) |
| Male | 1.0 (Reference) | **1.22 (1.03 - 1.46)** | **1.70 (1.09 - 2.63)** |
| Personal/family/friend diagnosis of COVID-19 | | | |
| No | 1.0 (Reference) | 1.0 (Reference) | 1.0 (Reference) |
| Yes | 1.0 (Reference) | **0.79 (0.58 - 1.06)** | **0.46 (0.17 - 1.29)** |
| Beliefs in support of the participation of medical students in COVID-19 pandemic healthcare | | | |
| S10. It is the duty of the medical student to put himself or herself at the service of the population in the pandemic | 1.0 (Reference) | **7.15 (5.85 - 8.74)** | **23.16 (13.60 - 39.41)** |
| S28. I am willing to take risks by participating in practice in the context of the pandemic | 1.0 (Reference) | **12.45 (10.24 - 15.14)** | **13.42 (7.79 - 23.13)** |
| S6. I am able to participate in the care of patients who seek health care | 1.0 (Reference) | **6.99 (5.82 - 8.41)** | **6.48 (3.92 - 10.72)** |
| S2. I can identify signs of severity in a patient | 1.0 (Reference) | **3.31 (2.48 - 4.41)** | **5.96 (1.87 - 19.01)** |
| S4. I know how to guide patients in therapeutic measures | 1.0 (Reference) | **2.71 (2.29 - 3.20)** | **4.78 (2.93 - 7.78)** |
| S22. I will be a better health professional for having experienced the pandemic | 1.0 (Reference) | **3.79 (3.19 - 4.50)** | **4.46 (2.72 - 7.31)** |
| S24. The supervision I receive in my practice fields is good | 1.0 (Reference) | **2.88 (2.44 - 3.41)** | **4.30 (2.70 - 6.85)** |
| S7. I feel able to communicate a diagnosis of COVID-19 infection | 1.0 (Reference) | **3.36 (2.83 - 3.99)** | **4.25 (2.58 - 7.03)** |
| S25. I have access to psychological support | 1.0 (Reference) | **2.21 (1.85 - 2.65)** | **3.57 (2.31 - 5.52)** |
| S5. I know how to use personal protection equipment (PFE) | 1.0 (Reference) | **2.48 (2.03 - 3.03)** | **2.76 (1.51 - 5.02)** |
| S3. I know how to guide patients in preventive measures | 1.0 (Reference) | **5.34 (3.11 - 9.17)** | 2.19 (0.68 - 7.08) |
| S26. I am proud of the way my institution responded to social and health demands in the face of the pandemic | 1.0 (Reference) | 1.14 (0.97 - 1.35) | **1.74 (1.13 - 2.68)** |
| S1. I feel prepared to identify a patient with suspected infection | 1.0 (Reference) | **2.72 (2.21 - 3.33)** | **1.72 (1.02 - 2.89)** |
| S19. I feel able to study my medical course content through distance learning | 1.0 (Reference) | 1.05 (0.89 - 1.24) | **1.70 (1.07 - 2.71)** |
| Beliefs not/poorly related with the participation of medical students in COVID-19 pandemic healthcare | | | |
| S17. After the pandemic, academic activities must be fully resumed | 1.0 (Reference) | **0.84 (0.71 - 0.98)** | **1.66 (1.07 - 2.58)** |
| S18. After the pandemic, only practical academic activities must be resumed | 1.0 (Reference) | 1.04 (0.88 - 1.23) | 1.40 (0.91 - 2.15) |
| S20. I prefer to study theoretical content using distance learning methods | 1.0 (Reference) | 0.86 (0.73 - 1.01) | 0.99 (0.64 - 1.52) |
| Beliefs against the participation of medical students in COVID-19 pandemic healthcare | | | |
| S13. Medical schools must suspend their academic activities during the first to fourth years. | 1.0 (Reference) | 0.78 (0.59 - 1.03) | **0.21 (0.13 - 0.35)** |
| S14. Medical schools must suspend their academic activities during internships | 1.0 (Reference) | **0.16 (0.13 - 0.19)** | **0.29 (0.19 - 0.46)** |
| S23. I feel stressed in the hospital at the moment | 1.0 (Reference) | **0.27 (0.22 - 0.32)** | **0.41 (0.27 - 0.64)** |
| S12. I am afraid of contaminating myself | 1.0 (Reference) | **0.35 (0.29 - 0.42)** | **0.50 (0.32 - 0.79)** |
| S27. The role of medical students during the pandemic is irrelevant | 1.0 (Reference) | **0.14 (0.10 - 0.20)** | 0.67 (0.36 - 1.24) |
| S21. My emotional state during the pandemic affects my learning | 1.0 (Reference) | **0.47 (0.39 - 0.55)** | 0.69 (0.45 - 1.07) |
| S11. I feel insecure regarding the future | 1.0 (Reference) | **0.68 (0.56 - 0.81)** | 0.98 (0.60 - 1.61) |
| S15. Distance learning must be implemented during the suspension of academic activities | 1.0 (Reference) | **0.83 (0.70 - 0.98)** | 1.00 (0.63 - 1.59) |
| S16. I would prefer to delay my training to fully replace academic activities than to participate in distance learning activities | 1.0 (Reference) | **0.68 (0.56 - 0.81)** | 1.02 (0.64 - 1.60) |
