# Supplementary material for: Medical students’ perceptions and motivations during the COVID-19 pandemic
Source: PLoS One. 2021 Mar 17;16(3):e0248627. doi: 10.1371/journal.pone.0248627 (PMC7968644; doi:10.1371/journal.pone.0248627)
Supplement: S1 Questionnaire — (PDF) [file pone.0248627.s006.pdf]

Medical Students during the COVID-19 pandemic

We invite you, a medical student, to participate in the research project "The medical student in the Covid-19 pandemic".

We, at the Center for Development of Medical Education of the School of Medicine of the University of Sao Paulo, developed this questionnaire to investigate the impacts of the Covid-19 pandemic on the experience of medical students worldwide and their perceptions. With this, we seek to generate scientific evidence to assess the effects of decisions by educational institutions and health authorities in the face of this pandemic.

By responding, you are giving your Free and Informed Consent for this survey.

To answer, use your mobile phone in a horizontal position.

We appreciate the participation and count on your help to broaden our results!

Biological sex:

Female

Male

Ages (in years)

In which city do you study?

In which institution do you study?

What year are you studying during medical graduation?

1st

2nd

3rd

4th

5th

6th

Other (enrollment locked, non-periodized, internship abroad)

Did your institution suspend all activities?

From first year to internship

Kept only medical internship

Not suspended

Have you ever been diagnosed (clinically or laboratory) with Covid-19 infection?

Yes

No

Has any familiar or friend of you ever been diagnosed (clinically or laboratory) with Covid-19 infection?

Yes

No

About the COVID-19 pandemic, answer: (Totally agree, Agree, Neither agree nor disagree, Disagree, Totally disagree)

1 I feel prepared to identify a patient with suspected infection.

2 I can identify signs of severity in a patient.

3 I know how to guide patients in preventive measures.

4 I know how to guide patients in therapeutic measures.

5 I know how to use personal protection equipment (PFE).

6 I am able to participate in the care of patients who seek health care.

7 I feel able to communicate a diagnosis of COVID-19 infection.

8 Medical internship students must participate in health care assistance during pandemic.

9 All students, regardless of their year in medical school, must participate in health care assistance during pandemic.

10 It is the duty of the medical student to put himself or herself at the service of the population in the pandemic.

11 I feel insecure regarding the future.

12 I am afraid of contaminating myself.

13 Medical schools must suspend their academic activities during the first to fourth years.

14 Medical schools must suspend their academic activities during internships.

15 Distance learning must be implemented during the suspension of academic activities.

16 I would prefer to delay my training to fully replace academic activities than to participate in distance learning activities.

17 After the pandemic, academic activities must be fully resumed.

18 After the pandemic, only practical academic activities must be resumed.

19 I feel able to study my medical course content through distance learning.

20 I prefer to study theoretical content using distance learning methods.
21 My emotional state during the pandemic affects my learning.
22 I will be a better health professional for having experienced the pandemic.
23 I feel stressed in the hospital at the moment.
24 The supervision I receive in my practice fields is good.
25 I have access to psychological support.
26 I am proud of the way my institution responded to social and health demands in the face of the pandemic.
27 The role of medical students during the pandemic is irrelevant.
28 I am willing to take risks by participating in practice in the context of the pandemic.
