# Supplementary material for: Medical students’ perceptions and motivations during the COVID-19 pandemic
Source: PLoS One. 2021 Mar 17;16(3):e0248627. doi: 10.1371/journal.pone.0248627 (PMC7968644; doi:10.1371/journal.pone.0248627)
Supplement: S2 Questionnaire — (PDF) [file pone.0248627.s007.pdf]

O estudante de medicina na pandemia de Covid-19

Convidamos você, estudante de medicina, para participar do projeto de pesquisa "O estudante de medicina na pandemia de COVID-19".

Nós, do Centro de Desenvolvimento de Educação Médica da Faculdade de Medicina da Universidade de São Paulo, desenvolvemos um questionário para investigar os impactos da pandemia de COVID-19 na vivência dos estudantes de medicina e suas percepções. Buscamos, com isso, gerar evidências científicas para avaliar os efeitos das decisões das instituições de ensino e das autoridades da saúde frente a essa pandemia.

Ao responder, você estará dando seu Consentimento Livre e Esclarecido para esta pesquisa.

Se puder, encaminhe para outro colegas estudantes de medicina.

Para responder, use seu celular na posição horizontal.

Agradecemos sua participação.

Sexo biológico
Feminino
Masculino

Idade (anos)

Em qual instituição você estuda?

Em que cidade você estuda?

Você está cursando qual ano da graduação de medicina?

1º

2º

3º

4º

5º

6º

Outro (matrícula trancada, desperiodizado, em estágio no exterior)

Sua instituição suspendeu todas as atividades?

Do primeiro ao sexto ano

Manteve somente o internato

Não suspendeu as atividades

Você já foi diagnosticado(a) (clínica ou laboratorial) com infecção por COVID-19?

Sim

Não

Algum familiar ou amigo seu já foi diagnosticado (clínica ou laboratorialmente) com infecção por COVID-19?

Sim

Não

Sobre a pandemia de COVID-19, responda

Concordo totalmente, concordo, não concordo nem discordo, discordo ou discordo totalmente

1. Sinto me preparado para identificar um caso suspeito
2. Consigo identificar sinais de gravidade em um paciente
3. Sei orientar medidas preventivas
4. Sei orientar medidas terapêuticas
5. Sei utilizar equipamentos de proteção individual

6. Estou apto a participar do atendimento dos pacientes que procurem o serviço de saúde
7. Sinto-me apto para comunicar o diagnóstico de infecção por covid-19
8. Estudantes de internato devem participar da assistência à saúde na pandemia
9. Todos os estudantes de medicina, em qualquer fase do curso, devem participar a assistência à saúde na pandemia
10. É dever do estudante de medicina se colocar à serviço da população na pandemia
11. Sinto-me inseguro em relação ao futuro.
12. Tenho medo de me contaminar.
13. O curso de medicina deve suspender as atividades acadêmicas do primeiro ao quarto ano
14. O curso de medicina deve suspender as atividades acadêmicas do internato
15. Ensino a distância deve ser implementado a partir da suspensão das atividades acadêmicas
16. Eu prefiro atrasar a minha formação para repor integralmente as atividades acadêmicas, do que participar das atividades de ensino à distância
17. Após a pandemia, as atividades acadêmicas devem ser repostas integralmente
18. Após a pandemia, somente as atividades práticas devem ser repostas.
19. Sinto-me apto a estudar os conteúdos do meu curso médico à distância
20. Prefiro estudar os conteúdos teóricos com métodos de ensino à distância
21. Meu estado emocional, frente a pandemia, prejudica o meu aprendizado
22. Serei um profissional de saúde melhor por ter vivenciado a pandemia
23. Sinto-me estressado no ambiente hospitalar no momento atual
24. A supervisão que recebo em campo de prática é boa.
25. Tenho acesso a suporte psicológico
26. Tenho orgulho da forma como minha instituição respondeu às demandas sociais e de saúde perante a pandemia.
27. O papel do estudante de medicina na pandemia é irrelevante
28. Estou disposto a correr riscos participando de atividades práticas no contexto da pandemia
