# Supplementary material for: Medical students’ perceptions and motivations during the COVID-19 pandemic
Source: PLoS One. 2021 Mar 17;16(3):e0248627. doi: 10.1371/journal.pone.0248627 (PMC7968644; doi:10.1371/journal.pone.0248627)
Supplement: Attachment — Submitted filename: responsetoreviewers.pdf [file pone.0248627.s009.pdf]

Manuscript PONE-D-20-15134

Medical student's perceptions and motivations in time of COVID-19 pandemic PLOS ONE

Response to Reviewers

For the convenience of the Editor and the Reviewers, we have retyped the questions and criticisms of the Reviewers.

Reviewer #1:

Important study, examining medical students' motives to participate in COVID19 emergency services. Valuable in looking at students as people with their own values, not just at available labor, and motivated by altruism, not just a wish to advance clinical knowledge. Well-designed and clearly presented.

Reviewer #2:

I want to start with a positive comment on the high return rate the authors received from the participants. I do think this is a strong research article; however, I would suggest the authors explore some analysis options to verify their assumptions.

My only major piece of feedback is to have the authors conduct a factor analysis of their predictor variables for a couple of reasons: 1. lesson the number of predictors in their logistic regression, and 2. Confirm that the variables measure the same underlying construct they hypothesize.

Response: Thank you for the suggestion. We included in the manuscript the factor analysis of the questionnaire.

We performed an exploratory factor analysis (EFA) using varimax rotation to identify underlying latent variables in our questionnaire. The criteria for EFA

model selection were (1) models with sum of squared loadings above 1 for all latent variables were initially considered and (2) among these models, we selected the model with the highest number of factors. This led to the selection of a model with four factors. Items with loadings below -0.3 or above 0.3 were considered as relevant for each factor. To compute the values for each latent variable, we included the loadings from all items, whether they met or not the criteria for relevance.

Factor analysis resulted in four domains: remote learning, medical knowledge self-efficacy, psychological stress and professional values.

We included in the supplementary material a new table, with the results of the factor analysis (Supplemental Table 2).

We also included the four domains in the regression analysis and included the results in Table 4.

On line 137, I would change the beginning of the sentence to read: The participants indicated their degree of agreement with ...

Response: we made this change in the manuscript.

This next comment may be a language difference, on page 15, line 167. I am unclear as to what "vacancies" means. Is it the number of non-respondents?

Response: Vacancies in the text mean number of positions for first-year medical students. We changed the text to make this point clear.

Page 27, line 312. I think using just the term "self-efficacy" is too broad within the context of the variables you used. I would add an adjective to better capture what you think the variables you used to contextualize them specific to the study. For example, clinical self-efficacy, or medical knowledge self-efficacy (a little wordy), etc.

Response: We agree and modified the text to “medical knowledge self-efficacy”.

Reviewer #3:

The area of research is relevant in the current healthcare landscape. The article is generally well written. However, a number of key components needed to assess scientific merit and infer findings need clarification and elaboration.

1. The aim of the study should be clearly specified. There are a number of tables presented - distribution by year of graduation, gender. The division into 3 groups and analysis thereof was particularly not clear to me. If the aims were clearly specified, it would make things easier for me to understand.

Response: The aim of our study was to evaluate motivation of medical students to be part of the heath team to help in the COVID-19 pandemic. To evaluate the motivation of medical students, we decided to develop a questionnaire specifically designed for this purpose.

To perform a regression analysis, we divided the medical students in three groups, according to their opinion regarding who, in their opinion, should participate in the care of patients with COVID-19. The three groups were divided considering the following:

a) Students that responded that medical students should not participate;
b) Students that responded that only students in their final years of medical school should participate (only interns);

c) Students the responded that all medical students should participate in the care of people with COVID-19 (all medical students).

2. The authors mention that the study questionnaire was specifically designed for this study. However, I would prefer to get more information on how it was designed. For example, who were the members of the panel and were the decision guided by public health needs, research questions, other similar surveys, etc. Whether any specific domains of interest were tested in these 28 statements. If not, adding some explanation on why as many attitudes would be important to study would be relevant.

Response: We agree and included in Methods a more detailed description of how the questionnaire was designed. After a careful search in the literature, with did not find any questionnaire designed to assess motivations of medical students to help in the care of people during a pandemic or any other health emergency. So, we decided to develop a new questionnaire.

We performed four meetings, two with medical students and two with faculty from the Center for Development of Medical Education of our medical school. Each meeting had 10-15 participants and lasted 2-3 hours. We asked the following questions to the participants:
“To assess motivations of medical students to work with the health team in the care of people with COVID-19, which questions should be asked?”
“What are the main concerns of medical students related to work with people with COVID-19?”
“What questions should be asked to evaluate the opinions and motivations of medical students concerning the need to move to on-line teaching due to the impact of COVID-19?”

We recorded the meetings, and three researchers wrote the questionnaire including all suggestions from the medical students and faculty meetings.

The questionnaire was then revised by a group of medical students and faculty, until a consensus was reached that all the statements were easy to understand.

A second revision was performed after a pilot application of the questionnaire to a group of twenty medical students.

We included this explanation in Methods

3. Response rates seem to be determined by the geography, with better response among southeast regions. Authors could comment on measures they took to ensure uniform response rates and possibly comment in the discussion on how representative their results might be.

Response: In Table 1 we show the number of respondents from each one of the five geographic regions of Brazil and show the proportion of respondents and number of positions for first-year medical students (vacancies), showing that there was a similar percentage of respondents from each one of the regions. The southeast region of Brazil is the region with more population and more medical schools. We agree that this was not clear in the text and we explained that “vacancies” were the number of first year positions offered to medical students.

4. Analysis is appropriate. However, it is necessary to state why the specific variables were selected for the regression analyses. Much of the rationale for grouping or conducting specific analyses are mentioned later on in the discussion. I feel this needs to be explained before the results to understand the significance of the presented data.

Comments: We included in regression models all 28 statements of the questionnaire as dependent variables (percentages of students that agreed with each one of the 28 statements). We controlled the analysis for sex, year of medical course and region of the country. We included this information in Methods.

We included the rationale for grouping and the analyses in the Methods section.

5. I found the tables rather lengthy although I understand that this is due to the length of the developed questionnaire. Perhaps the authors may want to be selective on the amount of information shared in tables within the manuscript and possibly as supplementary tables. Flow of text under results section needs to be improved.

Comments: we revised the flow of text under results section.

Regarding tables 3-5, I wasn't sure why the items are presented in the random sequence. It may be useful to keep to an ascending sequence based on items number to allow readers to easily compare the results.

Comments: The items in tables 3-5 are not presented in random sequence. In table 3 "Students characteristics and perceptions according to their view about the role of medical students during the COVID-19 pandemic", the items are presented in descending sequence concerning the percentage of agreement of each one of the statements of the entire sample of medical students. In tables 4 and 5 ("Adjusted odds ratios for the association between students' characteristics and perceptions and their view about the role of medical students during the COVID-19 pandemic" and "Adjusted odds ratios for the association between internship students' characteristics and perceptions and their view about the role of medical students during the COVID-19 pandemic"), the items are presented in descending sequence concerning the odds ratios comparing the group "all medical students should participate in the pandemic" to "no medical students should participate in the pandemic". We included this information in the Results section of the manuscript.

6. Discussion is generally well written. Authors however need to state limitations of their research and ways in which these might have influenced the findings.

I would urge the authors to look at standard guidelines, for example STROBE.

Response: We included a paragraph in Discussion about the limitations of our study. We designed our study and report according to STROBE guidelines for studies and included this information in the Methods section of the manuscript.
