# Supplementary material for: Medical students’ perceptions and motivations during the COVID-19 pandemic
Source: PLoS One. 2021 Mar 17;16(3):e0248627. doi: 10.1371/journal.pone.0248627 (PMC7968644; doi:10.1371/journal.pone.0248627)
Supplement: Attachment — Submitted filename: point_by_point_reply.docx [file pone.0248627.s010.docx]

Manuscript PONE-D-20-15134R1

“Medical students’ perceptions and motivations during the COVID-19 pandemic”

Point-by-point reply to the comments of Reviewer #3

Dear Dr. Janhavi Ajit Vaingankar

Academic Editor

PLOS ONE

Thank you for your letter with the invitation to submit a revised version of our manuscript addressing the points raised by reviewer #3.

For your convenience we have retyped the comments of the reviewers.

Reviewer #2: The authors addressed all my comments. They correctly pointed out that I made a mistake in my comments about factor analysis.

Reviewer #3: Authors seem to have addressed most of the suggestions. I would however like to provide few further comments for consideration:

1. Methods section could be made clearer by providing a proper flow to the content. Perhaps adding sub sections such as ethics, study setting, participant criteria, development of questionnaire and data collection could offer clarity to the readers.

Response: We added sub sections to Methods, as suggested: ethics, development of questionnaire, data collection, study variables and statistical analysis.

2. Statistical section: The factor structure was performed after suggestions in earlier reviewer. However, it is left at that. Authors should consider how the 4 factors could be used to score the responses and then use them to perform regression analysis for the subscales. The tables are currently very lengthy and far too much data is presented. The item-wise distribution does not add much value as there aren't many evident differences in the three groups. I would urge the authors to just focus on subscale scores for some of the analysis and their determinants instead of item score differences.

Response: We included the factor analysis in the revised version of the manuscript and included the four domains identified in the multinomial logistic regression models to study the association between the students’ characteristics and perceptions according to their opinion about student participation in the healthcare of the COVID-19 pandemic (Table 3: Adjusted odds ratios for the association between students’ characteristics and perceptions and their views on the role of medical students during the COVID-19 pandemic). We included both the four domains and all statements of the questionnaire in the logistic regressions.

To decrease the amount of data presented in the main manuscript we moved former table 3 (“Student characteristics and perceptions according to their views about the role of medical students during the COVID-19 pandemic”) to supplementary material.

3. The discussion needs to clearly address the aim of the study which was to identify motivations of the students to be part of pandemic teams. A summary of the main motivations or unexpected findings could be provided at the beginning. This can also help in understanding the multitude of results presented.

Response: We agree and included a new paragraph in the beginning of Discussion (paragraph #2) with the summary of the main motivations of medical students observed).
