# Supplementary material for: Medical students’ perceptions and motivations during the COVID-19 pandemic
Source: PLoS One. 2021 Mar 17;16(3):e0248627. doi: 10.1371/journal.pone.0248627 (PMC7968644; doi:10.1371/journal.pone.0248627)
Supplement: Attachment — Submitted filename: point_by_point_reply2.docx [file pone.0248627.s011.docx]

Manuscript PONE-D-20-15134R2

“Medical students’ perceptions and motivations during the COVID-19 pandemic”

Point-by-point reply to the comments of Reviewer #3

Dear Dr. Janhavi Ajit Vaingankar

Academic Editor

PLOS ONE

Thank you for your letter with the invitation to submit a revised version of our manuscript addressing the points raised by reviewer #3.

Reviewer #3: Authors have addressed my comments well. However, I have one further comment regarding the scores derived from the four factors. Authors should add how the scores were obtained (eg total scores, weighted scores etc) in the analysis section. This will make it easier to interpret the ORs presented in Table 3. I can understand the categorical responses for each item and the reference used therein. But I am not clear how from each factor (eg. altruism) a categorical response was obtained? I gather there were more than 1 item in each factor? Some clarity on this will be useful.

Answer to reviewer #3: Thank you for your suggestion. In the new version of the paper, we clarified the interpretation of odds ratios for exploratory factor analysis (EFA) latent variables. EFA latent variables are included in regression models as continuous independent variables. In the new version, to improve interpretation and comparability, we standardized the scores for each EFA latent variable, adopting a mean of zero and standard deviation of one. Therefore, the odds ratios presented in the paper relate to a 1 SD increase in the factor score.

We included in Methods:

For exploratory factor analysis (EFA) and regression models, agreement with each statement was considered as a dichotomous variable. In these cases, participants who answered “totally agree” or “agree” were considered as agreeing with each statement. We performed an EFA using varimax rotation to identify the underlying latent variables (factors) in our questionnaire. The criteria for EFA model selection were as follows: (1) models with sum of squared loadings greater than 1 for all latent variables were initially considered, and (2) among these models, we selected the model with the highest number of factors. This process led to the selection of a model with four factors. Items with loadings less than -0.3 or greater than 0.3 were considered relevant for each factor (Supplemental table 1). To compute each EFA latent variable score, we attributed weights for all items, according to their loadings in the final model, regardless of whether they met the criteria for relevance. After this procedure, we standardized the values to obtain a mean of zero and standard deviation (SD) of one for each factor score. Therefore, the odds ratios presented in these models relate to a 1 SD increase in the EFA latent variable score.
